# One Health: Eine Sichtweise des informellen ministeriellen Netzwerkes: „Was wäre, wenn der One-Health-Ansatz zum Leitmotiv von Kooperation auf nationaler, europäischer und globaler Ebene würde?“

**DOI:** 10.1007/s00103-023-03706-3

**Published:** 2023-05-19

**Authors:** Luzie Verbeek, Denise Rabold, Arne Hartig, Sophia Stephan, Elisabeth Deus, Insa Otte, Anne Beutling, Karoline Schollmeyer, Pieter de Coninck, Karin Höppner, Kristina Saal, Timo Vogler, Lukas Hach, Elke Steinmetz, Thomas Benner, Leonie Derksen, Nina Militzer, Carolina Probst, Ute Teichert

**Affiliations:** 1 Abteilung 6 „Öffentliche Gesundheit“, Referat 615 „One Health, Antimikrobielle Resistenzen“, Bundesministerium für Gesundheit, Mauerstr. 29, 10117 Berlin, Deutschland; 2 Bundesministerium für Gesundheit, Bonn, Deutschland; 3 Auswärtiges Amt, Berlin, Deutschland; 4 Bundeskanzleramt, Berlin, Deutschland; 5 Bundesministerium für Bildung und Forschung, Bonn, Deutschland; 6 Bundesministerium für Ernährung und Landwirtschaft, Bonn, Deutschland; 7 Bundesministerium der Justiz, Berlin, Deutschland; 8 Bundesministerium für Umwelt, Naturschutz, nukleare Sicherheit und Verbraucherschutz, Bonn, Deutschland; 9 Bundesministerium der Verteidigung, Bonn, Deutschland; 10 Bundesministerium für wirtschaftliche Zusammenarbeit und Entwicklung, Bonn, Deutschland

**Keywords:** Interdisziplinarität, Transformation, OHHLEP, Nachhaltigkeit, Gesundheitspolitik, Interdisciplinarity, Transformation, OHHLEP, Sustainability, Health policy

## Abstract

Aus Sicht der Bundesregierung ist der One-Health-Ansatz ein wegweisender Kompass für ein inter- und transdisziplinäres Denken, Vernetzen und Handeln. Zum Schutz der Gesundheit von Menschen, Tieren, Pflanzen und Ökosystemen sollte er stets an all seinen Schnittstellen und in allen Aktivitäten Beachtung finden. Der One-Health-Ansatz hat in den letzten Jahren an politischer Bedeutung gewonnen und wird in verschiedenen Strategien berücksichtigt.

In diesem Beitrag wird über die aktuellen Strategien mit One-Health-Ansatz berichtet. Dazu gehören u. a. die *Deutsche Antibiotika-Resistenzstrategie*, die *Deutsche Anpassungsstrategie an den Klimawandel*, die globale Initiative *Nature for Health* sowie das internationale Pandemieabkommen, das derzeit erarbeitet wird und in dem auch Prävention eine wichtige Rolle spielt. Die Themen Biodiversitätsverlust und Klimaschutz müssen in einen gemeinsamen Kontext gesetzt werden, der die Interdependenzen des Gesundheitszustandes von Menschen, Tieren, Pflanzen und Ökosystemen berücksichtigt. Indem relevante Disziplinen auf unterschiedlichen Ebenen selbstverständlich einbezogen werden, kann es gelingen, gemeinsam einen Beitrag zu einer nachhaltigen Entwicklung zu leisten, wie es die Agenda 2030 der Vereinten Nationen fordert. Diese Perspektive leitet das globale Engagement Deutschlands in der globalen Gesundheitspolitik hin zu mehr Stabilität, Freiheit, Vielfalt und Solidarität sowie Achtung der Menschenrechte – so kann ein holistischer Ansatz wie One Health zur Verwirklichung von Nachhaltigkeit und zur Stärkung demokratischer Prinzipien beitragen.

## Abstract (en)

In the view of the German government, the One Health approach is a pioneering compass for inter- and transdisciplinary thinking, networking, and action. To protect the health of humans, animals, plants, and ecosystems, it should always receive attention at all its interfaces and activities. The One Health approach has gained political importance in recent years and is being taken into account in several strategies.

This article reports on the current strategies using a One Health approach. These include the *German Antibiotic Resistance Strategy*, the *German Strategy for Adaptation to Climate Change*, the global initiative *Nature for Health*, and the international pandemic agreement, which is currently being drafted and in which prevention also plays an important role. The issues of biodiversity loss and climate protection must be placed in a common context that takes into account the interdependencies of the health status of humans, animals, plants, and ecosystems. By involving relevant disciplines at different levels as a matter of course, we can succeed in making a joint contribution to sustainable development, as required by the United Nations’ Agenda 2030. This perspective guides Germany’s global engagement in global health policy toward greater stability, freedom, diversity, solidarity, and respect for human rights. Thus, a holistic approach such as One Health can contribute to achieving sustainability and strengthening democratic principles.

## Verpflichtungen und Herausforderungen

Die 17 Ziele für nachhaltige Entwicklung (*Sustainable Development Goals, SDGs*), die im Jahr 2015 einstimmig in der Agenda 2030 [1] von den Vereinten Nationen beschlossen wurden, sind die Richtschnur des politischen Handelns der Bundesregierung [2]. Das Nachhaltigkeitsziel 3 (SDG 3) lautet: „Ein gesundes Leben für alle Menschen jeden Alters gewährleisten und ihr Wohlergehen fördern“. Dieses im Gesundheitssektor besonders präsente Ziel kann jedoch so wenig wie die anderen isoliert verstanden werden. Die 17 Nachhaltigkeitsziele können nicht einzeln, sondern nur gemeinsam erreicht werden. Die der Agenda 2030 zugrunde liegenden Aspekte *Mensch, Planet, Wohlstand, Frieden* und *Partnerschaft* sowie das Versprechen, auf dem Weg der Transformation zur Nachhaltigkeit niemanden zurückzulassen, verdeutlichen, dass alle Nachhaltigkeitsziele integriert und unteilbar sind (s. Infobox 1). Auf dem Weg zur Erreichung dieser Ziele gilt es daher, vielfältige Wechselwirkungen zu berücksichtigen, was eine ganzheitliche und sektorübergreifende Herangehensweise erfordert. So hängen z. B. die Folgen des Klimawandels mit seinen zunehmenden Extremwetterlagen nach heutigem Stand der Wissenschaft mit dem Verlust der Biodiversität zusammen. Beide Phänomene begünstigen das Auftreten von Gesundheitskrisen, darunter Pandemien, Hungerkrisen, Dürren, Migrationsbewegungen und Armut. Wenn der Klima- und Biodiversitätskrise nicht erfolgreich entgegengewirkt wird, wird sie mittel- und unmittelbar negative Auswirkungen auch auf die Gesundheit der Menschen haben.

## One-Health-Ansatz: Wegbereiter und Kompass

*One Health* ist ein Ansatz, der für ein Denken außerhalb fachspezifischer Silos steht und sich traditionell auf den Human- und Veterinärsektor bezog. Eine Weiterentwicklung erfuhr er in den „Berlin Principles“ zu One Health [3], die im Jahr 2019 im Auswärtigen Amt verabschiedet wurden und die die „Manhattan Principles“^1^ aus 2004 fortsetzen. Aktuell dient die One-Health-Definition des *One Health High Level Expert Panel *(OHHLEP; [4, 5]) als handlungsleitende Orientierung, wobei die Umweltaspekte explizit hervorgehoben werden (s. Infobox 2).

Die Bundesregierung nutzt den One-Health-Ansatz als gemeinsame Grundlage, um die hochkomplexen Phänomene und Herausforderungen unserer Zeit besser zu verstehen, Gesundheitsrisiken zu mindern und zukunftsweisende Lösungswege zu beschreiten. Im Fokus stehen hierbei die komplexen Zusammenhänge und gegenseitigen Abhängigkeiten zwischen der Gesundheit von Menschen und Tieren sowie gesunden und widerstandsfähigen Ökosystemen. In vielen Feldern ist die Berücksichtigung der Schnittstelle *Mensch-Tier-Pflanze-Umwelt* zentral, um effektive Maßnahmen zu entwickeln. Beispielhaft zu nennen sind hier Maßnahmen zur Minderung von Gesundheitsrisiken und gesundheitlichen Auswirkungen durch Biodiversitätsverlust und Klimawandel, zur Epidemie- und Pandemieprävention, zur Verhinderung antimikrobieller Resistenzen (AMR), zur Eindämmung vernachlässigter und armutsassoziierter Tropenkrankheiten und für eine verbesserte Lebensmittelsicherheit. One Health steht in diesem Prozess mit einem sektorübergreifenden und integrativen Management für eine „Ent-Siloisierung“ und kann als „way to live“ betrachtet werden, wie der Präsident des Friedrich-Loeffler-Instituts und Co-Chair vom OHHLEP, Prof. Dr. Dr. Thomas Mettenleiter, es auf verschiedenen Veranstaltungen zusammengefasst hat.

## Zunehmende politische Bedeutung von One Health

Der One-Health-Ansatz nimmt nicht nur in der Wissenschaft, sondern auch politisch eine zunehmend wichtige Rolle ein. Dies unterstreicht die Bundesregierung mit der *Strategie zur globalen Gesundheit* aus dem Oktober 2020 [6] sowie im aktuellen Koalitionsvertrag [2]. Zurückzuführen ist dieses Engagement einerseits auf die dringlichen globalen und existentiellen Krisen der Gegenwart, einschließlich der Rückschritte, die die Weltgemeinschaft bei der Erreichung der Nachhaltigkeitsziele durch die COVID-19-Pandemie erlitten hat, sowie andererseits auf die Einsicht, dass die Weiterverfolgung der bisherigen singulären Vorgehensweisen nicht zu tragfähigen Lösungen führen kann. National und international setzt die Bundesregierung auf eine Stärkung des One-Health-Ansatzes, um Synergieeffekte zu nutzen und widerstandsfähige Lösungen für die Anforderungen der Zukunft zu entwickeln. Während der deutschen G20-Präsidentschaft 2017 [7] haben sich die Gesundheitsministerinnen und -minister dazu verpflichtet, den One-Health-Ansatz in Kooperation mit zuständigen Organisationen der Vereinten Nationen zu stärken. Im Mai 2022 vereinbarten die G7-Gesundheitsministerinnen und -minister unter deutscher Präsidentschaft, die Beziehungen zwischen *Mensch-Tier-Pflanze-Umwelt* stärker in den Blick zu nehmen [8]. Der One-Health-Ansatz stellt so das verbindende Element zwischen den im Kommuniqué der G7-Gesundheitsministerinnen und -minister adressierten Themenfeldern *Pandemieprävention, -vorsorge* und *-reaktion, AMR* sowie *Klimaanpassung* und *Klimaschutz in Gesundheitssystemen* dar.

Wegweisend für eine noch intensivere Zusammenarbeit zwischen den Bundesministerien im Sinne von One Health ist die Gründung eines informellen One-Health-Netzwerks im Ressortkreis. Dieses entstand 2021 aus einem ressortübergreifenden strategischen Workshop zur Frage: „Was wäre, wenn der One-Health-Ansatz zum Leitmotiv von Kooperation auf nationaler, europäischer und globaler Ebene würde?“

## One Health konkretisiert sich

Das *One Health High Level Expert Panel *(OHHLEP; [9]) wurde im Mai 2021 auf deutsch-französische Initiative hin gegründet. Deutschland und Frankreich setzten sich zudem für die Verstetigung der Zusammenarbeit zwischen Weltgesundheitsorganisation (WHO), Weltorganisation für Tiergesundheit (WOAH), Ernährungs- und Landwirtschaftsorganisation der Vereinten Nationen (FAO) und dem Umweltprogramm der Vereinten Nationen (UNEP) im Bereich One Health ein, was im März 2022 zur Etablierung der „Quadripartite“ geführt hat. Ziel der Quadripartite ist die Optimierung der Gesundheit von Menschen, Tieren, Pflanzen und der Umwelt im Sinne des One-Health-Ansatzes. Als Expertengremium berät OHHLEP die Quadripartite zu One-Health-Themen, um die Koordinierung, Abstimmung und Priorisierung von internationalen Aktivitäten zu verbessern. Zu diesen Aktivitäten zählt u. a. das Mitwirken beim *One Health Joint Plan of Action (2022–2026) *der* Quadripartite *[10], ein konkreter Maßnahmenplan zur Implementierung von One Health (s. Infobox 3). Dieser wurde im Rahmen eines Side-Events des World Health Summit im Oktober 2022 im Museum für Naturkunde in Berlin offiziell präsentiert und wird von der Bundesregierung unterstützt.

## Anwendungsbeispiele für den One-Health-Ansatz

In Deutschland gibt es unterschiedliche Projekte, in denen die Bundesministerien das einschränkende Verständnis von Zuständigkeiten überwinden und das gemeinsame Handeln im Sinne des One-Health-Ansatzes optimieren.

Die Bundesregierung hat sich erfolgreich für die Entwicklung eines *Globalen Aktionsplans zur Bekämpfung von Antibiotika-Resistenzen* durch die WHO eingesetzt. Ein zentrales Element des Globalen Aktionsplans ist die Erstellung von nationalen Strategien zu antimikrobiellen Resistenzen (AMR). Seit 2008 erarbeitet die Bundesregierung ressortübergreifend die *Deutsche Antibiotika-Resistenzstrategie „DART“,* welche unter Berücksichtigung der Vorgaben des Globalen Aktionsplans darauf abzielt, noch vorhandene Lücken bei der Bekämpfung von Antibiotikaresistenzen in Deutschland zu schließen [11]. Essentiell ist dabei, dass die nationale AMR-Strategie nur Wirkung zeigen kann, wenn alle nationalen Akteure einen Beitrag zu ihrer Umsetzung leisten und ihre Aktivitäten an den Zielen der Strategie ausrichten. Dafür bietet auch der Austausch in der interministeriellen Arbeitsgruppe zu AMR seit Jahren eine wertvolle Gelegenheit.

Zur Anpassung an die klimatischen Veränderungen wurde bereits im Jahr 2008 die *Deutsche Anpassungsstrategie an den Klimawandel* (DAS; [12]) verabschiedet. Sie ist ein gelungenes Beispiel für eine ressortübergreifende Kooperation zu einem hochkomplexen Thema. In der interministeriellen Arbeitsgruppe zur DAS fließt fachliche Expertise aller Ressorts ein.

Eine *Lesson Learned* der COVID-19-Pandemie sind der Bedarf an einem verbesserten ressortübergreifenden Lagebild und die Notwendigkeit einer *One-Health-Surveillance*, durch die systematisch Daten verschiedener Bereiche besser als bisher geteilt, interpretiert und transparent gemacht werden. Dieser Ansatz des Wissensmanagements wird bereits vom Sanitätsdienst der Bundeswehr verfolgt, um so auch den multidisziplinären Herausforderungen der Biosicherheit gerecht zu werden [13]. Konkret wird bei der *abwasserbasierten Surveillance *[14] zu SARS-CoV‑2 eine technische und administrative Infrastruktur geschaffen und etabliert. Dies ermöglicht, dass Daten des Umweltbereiches einen Beitrag zum Lagebild im Gesundheitssektor leisten. So wird z. B. der Trend der Viruslast im Abwasser auf dem Pandemieradar des Robert Koch-Instituts visualisiert.

Auch in Entwicklungs- und Schwellenländern setzt sich die Bundesregierung für die Stärkung des One-Health-Ansatzes ein. So konnte beispielsweise gemeinsam mit UNEP und weiteren Partnern die neue globale Initiative „Nature for Health“ (N4H) mit einem Startkapital der Internationalen Klimaschutzinitiative (IKI; [15]) von 50 Mio. € als Multi-Partner-Trust-Fund ins Leben gerufen werden. N4H bringt führende Organisationen aus allen für One Health relevanten Bereichen zusammen, um das Risiko von Pandemien an möglichen Entstehungsorten zu reduzieren.

Vom 07.–19.12.2022 fand in Montreal, Kanada, die 15. Weltnaturkonferenz statt [16, 17]. Auch in diesem Zusammenhang wird deutlich, dass die Gesundheit des Menschen abhängig von der erfolgreichen Umsetzung der Ziele ist, die hierdurch direkt und indirekt One-Health-Aspekte adressieren. Letztlich müssen Klimawandel, Biodiversität und Gesundheit gemeinsam betrachtet werden [18, 19] und finden entsprechend auch im Rahmen der Weltklimakonferenzen Berücksichtigung.

Mit dem *Aktionsprogramm Natürlicher Klimaschutz* will die Bundesregierung nicht nur entscheidend dazu beitragen, die Ökosysteme in Deutschland zu verbessern, sondern es wird u. a. auch explizit auf die physische und psychische Gesundheit eingegangen: „Über die Schaffung von Naturerfahrungsräumen, urbanen Waldgärten und Wäldern sowie eine naturnahe Gestaltung und barrierefreie Erschließung von lokalklimatisch wirksamen Parkanlagen und Kleingewässern können neben der Verbesserung des Lokalklimas im direkten Wohnumfeld Räume für Bewegung, Erholung, Begegnung und insbesondere zur gesunden physischen und psychischen Entwicklung von Kindern entstehen“ [20]. Auch ohne explizite Erwähnung findet sich hier der One-Health-Ansatz und Co-Benefits werden zwischen natürlichem Klimaschutz und menschlicher Gesundheit verdeutlicht.

Im Dezember 2021 hat die Weltgesundheitsversammlung (WHA) in einer Sondersitzung beschlossen, einen Verhandlungsprozess für ein *internationales Pandemieabkommen* unter dem Dach der WHO einzuleiten [21]. Die COVID-19-Pandemie hat deutliche Barrieren in der globalen Pandemievorsorge und -reaktion aufgezeigt, die abgebaut werden müssen. Das neue, rechtsverbindliche Instrument, das die Internationalen Gesundheitsvorschriften (IGV) ergänzen soll, soll sich auf Pandemieprävention, -vorsorge und -reaktion konzentrieren. Im Pandemieabkommen setzt sich die Bundesregierung dafür ein, dass eine Form der Prävention adressiert wird, welche die Risiken eines Pathogen-Spillovers von Tier zu Mensch in den Fokus setzt. Hierbei wird die Verhinderung von Krankheitsausbrüchen, auch unter Berücksichtigung des One-Health-Ansatzes, grundsätzlich betrachtet. Um eine gemeinsame Linie zur Verankerung und Diskussion des One-Health-Ansatzes im Pandemieabkommen zu finden, luden das Auswärtige Amt und das Bundesministerium für Gesundheit im Februar 2023 zu einem ressortübergreifenden Workshop ein. Deutschland und die Europäische Union (EU) setzen sich zudem dafür ein, dass auch die Bekämpfung von Antibiotikaresistenzen – manchmal auch als *stille Pandemie* bezeichnet – im Pandemieabkommen angemessen geregelt wird. Laut WHA-Beschluss vom Dezember 2021 soll bei der 77. WHA im Mai 2024 ein Verhandlungsergebnis vorgelegt werden. Deutschland und die EU haben die Idee eines Pandemieabkommens von Anfang an befürwortet und unterstützt.

## One Health: Ausblick und Weiterentwicklung

Durch eine stärkere Einbeziehung wissenschaftlicher Erkenntnisse, insbesondere zur guten Anwendungspraxis des One-Health-Ansatzes, soll künftig die Arbeit der Bundesministerien fundiert weiterentwickelt werden. Hierfür ist auch eine entsprechende Forschungsförderung von Bedeutung. Einer Vereinbarung von 2016 zwischen den Bundesministerien für Bildung und Forschung (BMBF), Ernährung und Landwirtschaft (BMEL), Gesundheit (BMG) und Verteidigung (BMVg) haben sich 2022 zusätzlich die Bundesministerien für Umwelt, Naturschutz, nukleare Sicherheit und Verbraucherschutz (BMUV) und für wirtschaftliche Zusammenarbeit und Entwicklung (BMZ) angeschlossen und mit der *Forschungsvereinbarung One Health *[22] den Grundstein für eine noch stärkere ganzheitliche Betrachtung der Wechselwirkungen zwischen *Mensch-Tier-Pflanze-Umwelt* in Forschungsprojekten verfestigt – national wie auch international. Die Umsetzung von One Health wird sich im Sinne einer Querschnittsaufgabe auch im Öffentlichen Gesundheitsdienst (ÖGD) und Veterinärwesen sowie in den Umweltämtern wiederfinden. Beispiele der Zusammenarbeit gibt es bereits über die nationale Forschungsplattform für Zoonosen [23], die ressortübergreifend zu einer Forschungsplattform One Health weiterentwickelt werden soll [24].

Mit dem One-Health-Ansatz können wissenschaftsbasiert Hindernisse ressortübergreifend angegangen, Zielkonflikte austariert, Synergieeffekte genutzt, Wirkungen verstärkt und Kosten reduziert werden. Eine ressortübergreifende Surveillance wird hierbei für den Public-Health-Bereich eine zunehmend wichtige Rolle spielen.

Aber auch in Hinblick auf weitere aktuelle und zukünftige Herausforderungen kann der ganzheitliche Ansatz von One Health dazu dienen, die strukturelle Zusammenarbeit innerhalb der Bundesregierung zu stärken, indem er ein gemeinsames und integratives Verständnis für ein inter- sowie transdisziplinäres Zusammenarbeiten entwickelt und ermöglicht. Dabei ist zu betonen, dass ähnliche Ansätze (z. B. Planetary Health) aus Sicht der Bundesregierung synergistisch zu verstehen sind. Vergleichbare Entwicklungen gibt es im Bereich Nachhaltigkeit. Im Rahmen der *Deutschen Nachhaltigkeitsstrategie *(DNS; [25]) werden entsprechend dem Grundsatzbeschluss 2022 [26] zur Unterstützung der Umsetzung von Nachhaltigkeitsprinzipien und -zielen der Agenda 2030 in Transformationsbereichen ressortübergreifendes Denken und Wirken initiiert. Sogenannte Transformationsteams werden ressortübergreifend gemeinsame Themenschwerpunkte und Schnittstellen identifizieren und diese – ähnlich dem übergreifenden One-Health-Ansatz – über den eigenen Zuständigkeitszuschnitt hinaus betrachten und Formen der Kooperation entwickeln.

## „Was wäre, wenn der One-Health-Ansatz zum Leitmotiv von Kooperation auf nationaler, europäischer und globaler Ebene würde?“

Der One-Health-Ansatz bereitet mit seiner holistischen Betrachtung und Arbeitsweise einen Weg für die dringend notwendige Transformation zur Nachhaltigkeit. Als Leitmotiv von Kooperation auf nationaler, europäischer und globaler Ebene kann dieser holistische Ansatz zur Umsetzung von Stabilität, Freiheit, Vielfalt und Solidarität und zur Achtung der Menschenrechte beitragen, indem er eine gemeinsame sektoren- und disziplinübergreifende Arbeitsweise und entsprechend koordiniertes Handeln befördert. Von dieser Haltung – oder „way to live“ – hängt ab, wie erfolgreich wir die für unsere Demokratie und (globalen) Partnerschaften grundlegenden Ziele umsetzen und inwieweit wir den Herausforderungen unserer Zeit begegnen werden.

### Infobox 1 Die Agenda 2030 der Vereinten Nationen [1] …

- besteht aus 17 Nachhaltigkeitszielen, den Sustainable Development Goals (SDG), der Vereinten Nationen aus dem Jahr 2015,
- basiert auf 5 Kernaspekten „Mensch, Planet, Wohlstand, Frieden und Partnerschaft“, den sogenannten 5 „Ps“ für engl. *People, Planet, Prosperity, Peace, Partnership*, welche in den Nachhaltigkeitszielen miteinander verbunden sind,
- beruht auf dem Prinzip, niemanden zurückzulassen („leave no one behind“),
- zielt zum Beispiel auf die Bekämpfung von Hunger (SDG 2) und Armut (SDG 1), auf den Zugang zu sauberem Wasser (SDG 6) und auf Gesundheit und Wohlergehen für alle (SDG 3),
- soll alle Staaten darin unterstützen, die wesentlichen Ziele für alle Menschen und zukünftige Generationen zu erreichen und zu bewahren, und ist Grundlage dafür, Maßnahmen einzuleiten, die die Welt transformieren,
- soll die Transformation bis 2030 umsetzen.

### Infobox 2 One-Health-Definition des *One Health High Level Expert Panel* (OHHLEP; [4, 5])

(Vorläufige Übersetzung ins Deutsche nach T. Mettenleiter)

„One Health ist ein integrierter, vereinender Ansatz, der darauf abzielt, optimale und nachhaltige Gesundheit von Menschen, Tieren und Ökosystemen zu erreichen. Er erkennt an, dass die Gesundheit von Menschen, Haus- und Wildtieren, Pflanzen und der weiteren Umwelt (unseren Ökosystemen) eng miteinander verbunden und voneinander abhängig sind. Der Ansatz mobilisiert mehrere Sektoren, Disziplinen und die Bevölkerung auf allen Ebenen der Gesellschaft, um gemeinsam gegen Bedrohungen der Gesundheit und der Ökosysteme vorzugehen und gleichzeitig unseren kollektiven Bedarf an gesunden Lebensmitteln, reinem Wasser, nachhaltig erzeugter Energie und sauberer Luft zu decken, Maßnahmen gegen den Klimawandel zu ergreifen und eine nachhaltige Entwicklung zu fördern.“

### Infobox 3 One Health Joint Plan of Action (2022–2026)

Der „One Health Joint Plan of Action (2022–2026) – Working Together for the Health of Humans, Animals, Plants and the Environment“ wurde von den Quadripartite-Organisationen Welternährungsorganisation (FAO), Weltgesundheitsorganisation (WHO), Umweltprogramm der Vereinten Nationen (UNEP) und Weltorganisation für Tiergesundheit (WOAH, gegründet als OIE) veröffentlicht. Die Vision ist eine Welt, die besser in der Lage ist, Gesundheitsbedrohungen zu verhindern, vorherzusagen, zu erkennen und so zu reagieren, dass die Gesundheit von Menschen, Tieren, Pflanzen und der Umwelt verbessert und nachhaltig entwickelt wird. Mit dem Aktionsrahmen wird zur Erreichung der Ziele ein Maßnahmenprogramm zur Unterstützung angeboten. Es basiert auf der *Theory of Change*, um bestehende Barrieren abzubauen.

Inhaltlich werden 6 Action-Tracks formuliert:

1. Ausbau der One-Health-Kapazitäten zur Stärkung der Gesundheitssysteme
2. Verringerung der Risiken durch neu auftretende und wieder auftretende zoonotische Epidemien und Pandemien
3. Bekämpfung und Eliminierung endemischer Zoonosen, vernachlässigter tropischer Erkrankungen und vektorübertragener Erkrankungen
4. Stärkung der Bewertung, des Managements und der Kommunikation von Lebensmittelsicherheitsrisiken
5. Eindämmung der stillen Pandemie antimikrobieller Resistenzen
6. Integration der Umwelt in One Health

## Footnotes

1 https://oneworldonehealth.wcs.org/About-Us/Mission/The-Manhattan-Principles.aspx (zuletzt abgerufen am: 19.04.2023).
